# Lysophosphatidic acid down-regulates human *RIPK4* mRNA in keratinocyte- derived cell lines

**DOI:** 10.1371/journal.pone.0287444

**Published:** 2024-04-17

**Authors:** Lei Xu, Peter Bajorski, Brian Poligone

**Affiliations:** 1 Rochester General Hospital Research Institute, Cancer Biology Research, Rochester, New York, United States of America; 2 School of Mathematical Sciences, Rochester Institute of Technology, Rochester, New York, United States of America; University of Tennessee Health Science Center, UNITED STATES

## Abstract

The tight control of proliferating keratinocytes is vital to the successful function of the skin. Differentiation of dividing cells is necessary to form a skin barrier. The same dividing cells are necessary to heal wounds and when malignant form tumors. RIPK4, a serine-threonine kinase, plays critical roles in these processes. Its loss of function was associated with pathological keratinocyte proliferation and development of squamous cell carcinoma (SCC) in humans and mice. The current study extends previous findings in the importance of RIPK4 in keratinocyte proliferation. A serum-derived phospholipid, lysophosphatidic acid (LPA), was identified as an important biologic inhibitor of RIPK4. LPA functions by inhibiting the transcription of *RIPK4* mRNA. LPA treatment led to increased keratinocyte proliferation, and this was compromised in cells with reduced RIPK4 expression. The current study may help to explain the mechanism by which RIPK4 was downregulated during SCC progression and provide insights on RIPK4 functions. It may also allow for targeting of RIPK4 through a natural component of serum.

## Introduction

Keratinocyte proliferation is a vital process for skin development, wound healing, and skin tumor formation [1]. Its dysregulation has a variety of pathological consequences and is observed in psoriasis, defective wound healing, and tumorigenesis. Keratinocyte proliferation is regulated by a myriad of factors and signaling pathways, including Wnt, TP63, TGF-beta, NFBκB, and protein kinase C-mediated pathways.

The receptor interacting protein kinase 4 (RIPK4), also known as the protein kinase C-associated kinase (PKK), is a serine-threonine kinase acting downstream of protein kinase C (PKC) and mediates its activation partly through NF kappa B (NF-κB) [2–4]. RIPK4 has been shown to play critical roles in skin development and carcinogenesis [4]. Mutations in RIPK4 were found in patients with Popliteal Pterygium syndrome and Bartsocas-Papas syndrome [5, 6], a group of genetic disorders related to defective skin differentiation and fusion. Consistent with its role in skin development, germline deletion of *ripk4* in mice resulted in embryonic lethality with severe epidermal defects [7], whereas keratinocyte-specific deletion of *ripk4* led to thickened epidermis and impaired skin barrier formation in mice [8, 9]. Knockdown of *RIPK4* mRNA by RNAi in keratinocytes resulted in increased tumor size in xenografted transplants, supporting a tumor suppressor function for RIPK4 in skin keratinocytes [10, 11]. Keratinocyte eratinocyte-specific *ripk4-/-* mice also exhibited elevated skin tumorigenesis [9, 10], resonating its role as a tumor suppressor in squamous cell carcinoma (SCC) [10, 11]. Indeed, *RIPK4* mRNA was down-regulated in a majority of cutaneous SCC lines compared with normal human skin [11].

How *RIPK4* mRNA is down-regulated in SCC is unknown. Mutations in *RIPK4* were found in SCC samples, which were mostly missense mutations or targeting the functional kinase domain [12–14]. It is unclear if these mutations contribute to the lower level of *RIPK4* mRNA observed in SCC. While statistically significant, the occurrence of mutated *RIPK4* is relatively rare in SCC [12–14]. Therefore, it is conceivable that in most SCCs, the reduction in *RIPK4* mRNA is independent of its mutations but at the level of transcriptional regulations.

Adams et al. reported that serum treatment led to reduction of *RIPK4* mRNA in HaCaT cells [15], an immortalized keratinocyte cell line. Keratinocytes could be exposed to serum during wounding or in a tumor, where breakage of blood vessels leads to release of serum. As such, the down-regulation of *RIPK4* could be a consequence of wounding and/or tumorigenesis. Indeed, down-regulation of *RIPK4* was found in skin wounds in mice compared with normal skin [15] and SCC from humans express lower levels of *RIPK4* than adjacent normal keratinocytes [11]. Nevertheless, the factor in serum that induces the down-regulation of *RIPK4* may be available in other tissues and/or under different pathophysiological states. Identifying this factor is thus important for a complete understanding of RIPK4 regulation and its biological function.

In the current study, we investigated the factor(s) in serum that lead to down-regulation of *RIPK4* in the HaCaT cell line and multiple SCC cell lines. Through a variety of fractionation and biochemical approaches, we found that this factor was heat- and proteinase K-resistant and was not sensitive to DNase or RNase treatment. Our further characterization revealed that it was a phospholipid, lysophosphatidic acid (LPA). In serum, LPA is delivered by albumin as a carrier [16, 17]. LPA is also produced by resident tissues and cells and its level often elevated in pathological conditions [18–20]. LPA has been shown extensively to play pleotropic roles in biological processes, including angiogenesis, cell proliferation and migration, and contributes to a variety of diseases such as cancer, wound healing, and neurological impairments [21]. In our study, we show that LPA promoted cell cycle progression of the HaCaT cell line and the SCC cell line, A431, at least partly via down-regulating *RIPK4*. We hypothesize that the release of LPA during wounding or other pathological condition results in reduction of *RIPK4* in keratinocytes, contributing to keratinocyte proliferation.

## Materials and methods

### Reagents

Fetal bovine serum (FBS) was purchased from R&D Systems (cat# S11550) and FB Essence (FBE) was purchased from VWR (cat# 10803–034). Human plasma was provided by Dr. Michael Pichichero. Bovine serum albumin (BSA) was purchased from Fisher Scientific (cat #BP1600-100) and fatty acid-free BSA (FAF-BSA) was from Sigma (cat#126575). Proteinase K was from Takara Bio (cat#ST3041). Actinomycin D was from Fisher Scientific (#NC9856244). Lysophosphatidic acid (#L7260), Lysophosphatidylcholine (#L1881), Propidium Iodide (#P4170), and Centrifugal Filter Units (#UFC501008, #UFC505008, # UFC510008) were from Sigma. The MTT Cell Growth Assay Kit was from Fisher Scientific (#CT01).

### Cell lines

The HaCaT cell line was a gift from Dr. Lisa Delouise (University of Rochester Medical Center). The A431 cell line was purchased from ATCC (cat# CRL-1555). Both cell lines were cultured in DMEM (Fisher Scientific #11-960-069) plus 10% FBE and antibiotics. The SCC-1 cell line was provided by Dr. Alon Mantel and was cultured in keratinocyte serum-free medium with supplements (Fisher Scientific #17005042).

### Treatment of cells

Equal number of HaCaT or A431 cells were plated in each well of a 24-well plate. When they became confluent, the cells were starved overnight in serum-free DMEM. To test the effects of various reagents on the *RIPK4* mRNA level in cells, 50 μl of indicated reagent or PBS was added to 500 μl of serum-free DMEM in each wells, and cells were lysed two hours later (unless indicated otherwise) for RNA extraction using RNeasy Mini Kit (Qiagen, #74104). Each condition was tested in duplicates. To test the effects of heated FBS, FBS was heated at 95°C for one hour and then cooled on ice before administration to cells. Proteinase K digestion was performed by incubating FBS or 5% BSA or PBS with 1/40 volume of proteinase K at 56°C overnight. The mixture was subsequently heated at 95°C for 30 min to inactivate proteinase K followed by cooling on ice. The contribution of RNA or DNA in serum was examined by incubating FBS or PBS with 10 μg/ml DNase I or RNase A at room temperature for 30 min prior to administration to cells. To assess the transcription of *RIPK4* mRNA, 10 μg/ml Actinomycin D was added to HaCaT cells one hour prior to the addition of PBS and RNA was harvested two hours later.

### Quantitative RT-PCR

cDNA was synthesized from the RNA preparations using the Maxima H Minus First Strand cDNA Synthesis Kit (Fisher Scientific, #K1651). The Level of *RIPK4* mRNA and *GAPDH* mRNA (as control) was measured by qPCR using the PowerTrack Sybrgreen master mix (Fisher Scientific #A46109) and human *RIPK4*- or *GAPDH*-specific primers. The upstream primer for human *RIPK4* is 5’-CAGAAGAAGCCGTTTGCAGAT-3’ and downstream primer is 5’-GAGGCGTATCAGGTGGCTG-3’. The alternative primers were 5’- GATCTCCGGTTCCGAATCATC-3’ as the upstream primer and 5’-TCAGAAATCTTGACGTGGTAGTG-3’ as the downstream primer. The upstream primer for human *GAPDH* is 5’-GAAATCCCATCACCATCTTCCAGG-3’ and downstream primer is 5’-GAGCCCCAGCCTTCTCCATG-3’. The PCR was run in duplicates and analyzed in Bio-Rad CFX Connect Real-time PCR System. The average CT value of each duplicate was used to calculate relative mRNA levels of *RIPK4* mRNA, after normalization against one of the control samples, which was arbitrarily set to 1.

### Size-exclusion filtration

To assess the molecular weight of the serum factor that induces the down-regulation of *RIPK4* mRNA in human keratinocyte cell lines, 500 μl of FBS or FBE was added to Amicon Ultrafiltration columns with 10KD, 50KD, or 100KD cutoffs (Sigma-Aldrich #UFC501008, UFC505008, or UFC510008 respectively). After centrifugation at 14,000xg for 15 min at 4°C, the flow-through was collected, which supposedly contains molecules smaller than the cutoff. The solution retained in the column had a smaller volume than the original material, which supposedly contain molecules larger than the cutoff. This was conducted based on the manufacturer’s instruction and subsequently diluted to the original 500 μl with PBS before usage. The above collected fractions from columns with 100KD cutoff were analyzed on 7% SDS-polyacrylamide gel, followed by staining with SimplyBlue SafeStain (Fisher Scientific, cat# LC6060).

### *RIPK4* knockdown

*RIPK4* was knocked down in HaCaT and A431 cells by two different shRNAs, as described previously [22]. A scrambled shRNA was used as the control. The shRNAs were cloned into the pLKO.puro lentiviral vector (addgene.org) and virus was produced in HEK293 cells, according to established methods [11]. After infection, HaCaT or A431 cells were selected under 1 μg/ml puromycin and resistant populations were amplified and used for further analyses.

### Cell cycle analyses

Control or sh*RIPK4* cells were plated at 5x10^4^ (for HaCaT cells) or 1x10^5^ (for A431cells) per well in quadruplicates in 24-well plates. 24 hours later, the cells were starved in serum-free media prior to the treatment with 10 μM LPA. The vehicle FAF-BSA was used as control. The day after treatment, cells were harvested and fixed in cold 70% ethanol, followed by treatment with 10 μg/ml RNase A and 20 μg/ml propidium iodide, based on established protocols [23, 24]. Flow cytometry analyses were performed on LSRII instrument, using FACSDiva software.

### Western blot analyses

Cells were lysed in cold homogenization buffer (10 mM Tris-HCl, pH 8.0, 60 mM KCl, 1 mM EDTA, 1 mM DTT, 1% Triton X-100). The lysates were separated on SDS-polyacrylamide gel and probed with rabbit anti-RIPK4 antibody (Cell signaling #12636) and mouse anti-tubulin antibody (Sigma #T8328), followed by HRP-conjugated secondary antibodies. The chemiluminescence signals were detected by Clarify Western ECL substrate (Bio-Rad #170–5060) and captured by FluoChem E System (ProteinSimple).

#### MTT cell proliferation analysis

The MTT cell proliferation analyses were performed using the MTT Cell Growth Assay Kit, following the manufacturer’s instructions with some modification. Briefly, 20,000 HaCaT cells expressing control or shRIPK4 were plated in each of six wells of a 96-well plate overnight. They were then starved for 24 hours followed by treatment with vehicle (0.5% FAF-BSA) or 10 μM LPA. 10 μl of 5mg/ml MTT (3-(4,5-dimethylthiazol-2-yl)-2,5-diphenyltetrazolium bromide) was added to each well and, after 2 hrs of incubation, solubilized by 10% SDS/0.01N HCl. Absorbance at 570nm was measured by a plate reader (SpectraMax, Molecular Devices).

### Data processing and statistics

Results from multiple independent experiments were pooled, as indicated in figure legends. The mean was presented as bar graphs with standard deviation as the error. Student’s t-test was performed for data comparisons. A *p*-value less than 0.05 was considered statistically significant.

## Results

### Fetal bovine serum but not scratch wounding induced down-regulation of *RIPK4* mRNA in human keratinocyte-derived cell lines

**Fig 1:**
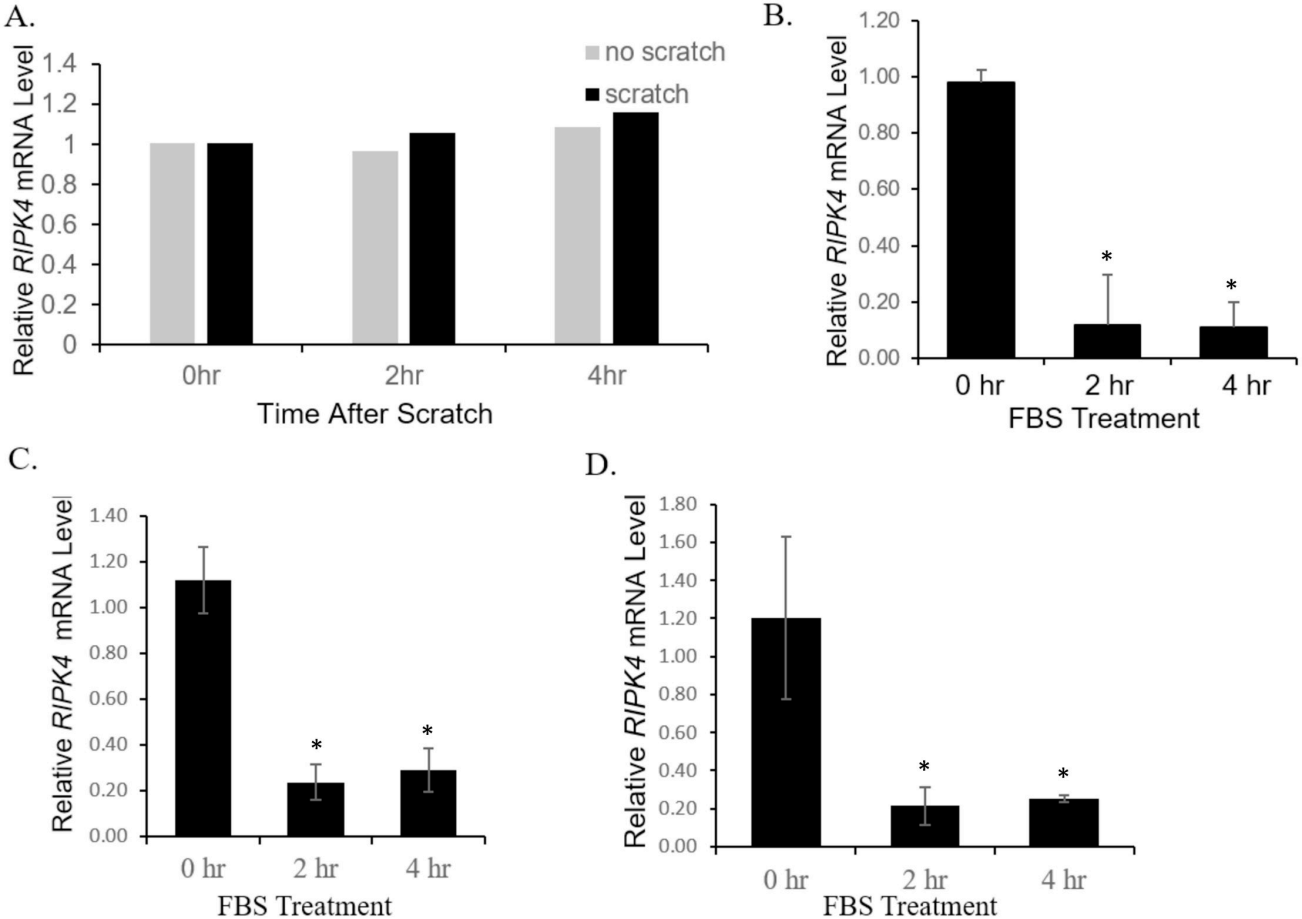
FBS treatment but not scratch wounding induced down-regulation of *RIPK4* mRNA in human keratinocyte-derived cell lines. A: A scratch was introduced in serum-starved HaCaT monolayer. The level of *RIPK4* mRNA was analyzed at 0, 2, or 4 hrs afterwards and did not differ from the unscratched cells. B-D: The level of *RIPK4* mRNA in HaCaT cells (B), A431 cells (C) or SCC-1 cells (D) was found reduced 2 hrs or 4 hrs after FBS treatment. Data in B were pooled from three independent experiments, data in C from two independent experiments, and data in D from two independent experiments for the 2 hr time point and one experiment for the 4 hr time point. *: *p* < 0.05.

*RIPK4* mRNA was reported to decrease in levels during wounding in mouse skin [15]. The mechanism of this down-regulation was investigated *in vitro* using the immortalized but non-tumorigenic keratinocyte cell line, HaCaT. The authors found that scratch wounding led to a reduction in *RIPK4* mRNA in these cells, as did serum and some growth factors. We were intrigued by these findings, since they implied, during the wounding process, both the mechanical tearing and excessive availability of serum and growth factors may contribute to the down-regulation of *RIPK4* in epidermis. We examined whether this was the case. A confluent but serum-starved HaCaT cell monolayer was subjected to scratch wounding and *RIPK4* mRNA levels were measured at different time points. In contrast to the report, we did not observe any effects of scratching on the level of *RIPK4* mRNA in HaCaT cells, either in the absence of serum (Fig 1A) or the presence of serum (S1A Fig). However addition of fetal bovine serum (FBS) to serum-starved HaCaT cells, led to a robust reduction in *RIPK4* mRNA level (Fig 1B) after two hours of incubation, consistent with the previous report [15]. We extended the analysis to two tumorigenic keratinocyte cell lines, A431 and SCC-1, and found that the reduction of *RIPK4* mRNA by FBS was recapitulated in these lines (Fig 1C and 1D). We also confirmed that serum treatments causes reduction of RIPK4 protein in human epithelial keratinocytes (HEKs) (S1B Fig., left). To confirm that the observed knockdown was specific to the *RIPK4* gene, we used an alternative pair of *RIPK4* primers that cover a different region of the *RIPK4* gene for the qPCR analyses. Similar down-regulation of *RIPK4* was observed after FBS treatment (S2 Fig).

The observed reduction in *RIPK4* mRNA level could be due to inhibition of nascent transcription of *RIPK4* or diminished stability of its mRNA. We investigated this by treating cells with actinomycin D, which inhibits nascent RNA transcription. Our result showed that actinomycin D treatment almost completely depleted *RIPK4* mRNA within the two hour of time-frame of analyses (S3 Fig), indicating that the regulation of *RIPK4* mRNA we observed was most likely due to impaired nascent transcription. Taken together, the above results suggest that FBS contains a factor that down-regulates *RIPK4* transcription in keratinocytes. We denote this factor “the serum factor” for the remaining manuscript.

**Fig 2:**
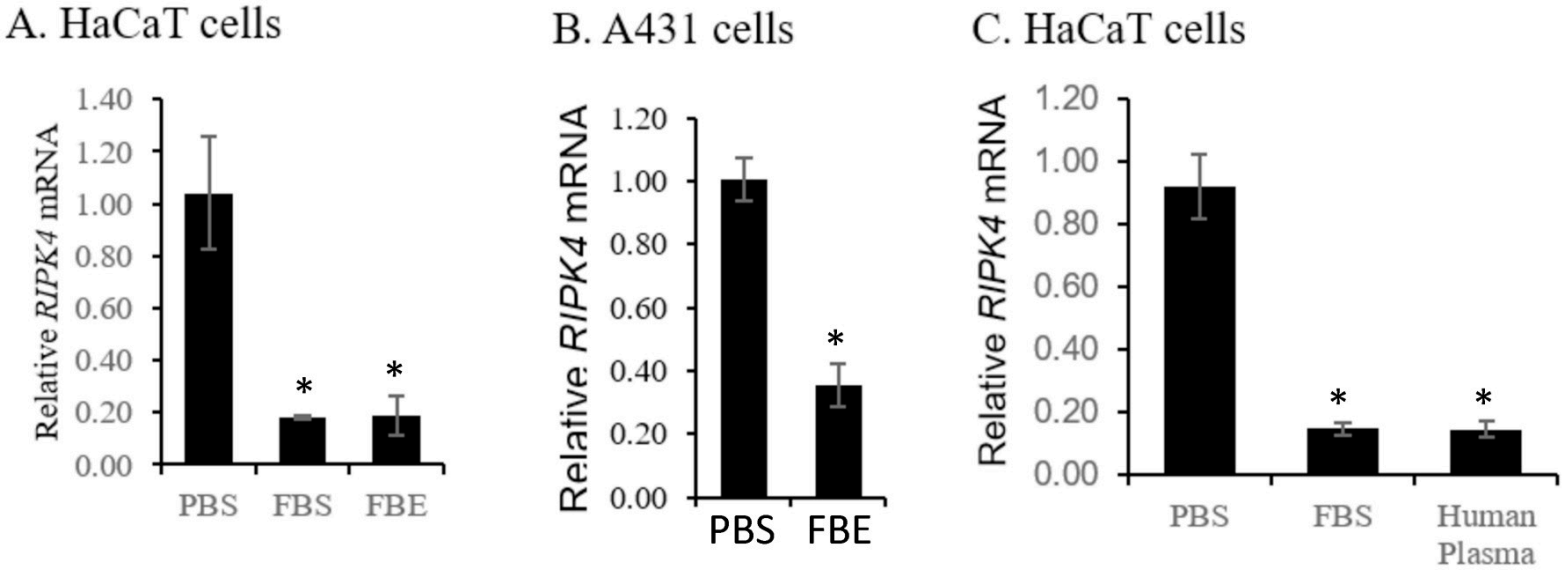
*RIPK4* mRNA was down-regulated by fetal bovine serum from different sources and by human plasma. A, B) The level of *RIPK4* mRNA in HaCaT (A) or A431 (B) cells was reduced after treatment by FBE and FBS. Data for FBE treatment were pooled from three independent experiments.C. The level of *RIPK4* mRNA in HaCaT cells was reduced after treatment of human plasma, similar to FBS. *: *p* < 0.05.

We then asked whether the observed effects were specific to the particular type of FBS. FB essence (FBE) is a cost effective alternative to FBS and contains a mixture of FBS, Bovine Calf Serum, Equine Serum, and a proprietary blend of supplements, vitamins, minerals, and growth factors. Its addition to cells induced a similar reduction in *RIPK4* mRNA level in HaCaT and A431 cells (Fig 2A and 2B). Importantly, human plasma, which would be most relevant to human skin biology, induced a robust reduction in *RIPK4* mRNA level in HaCaT cells (Fig 2C), similar to observation from FBS, arguing strongly that a serum factor may down-regulate *RIPK4* mRNA in human epidermis.

### Is the serum factor a protein?

**Fig 3:**
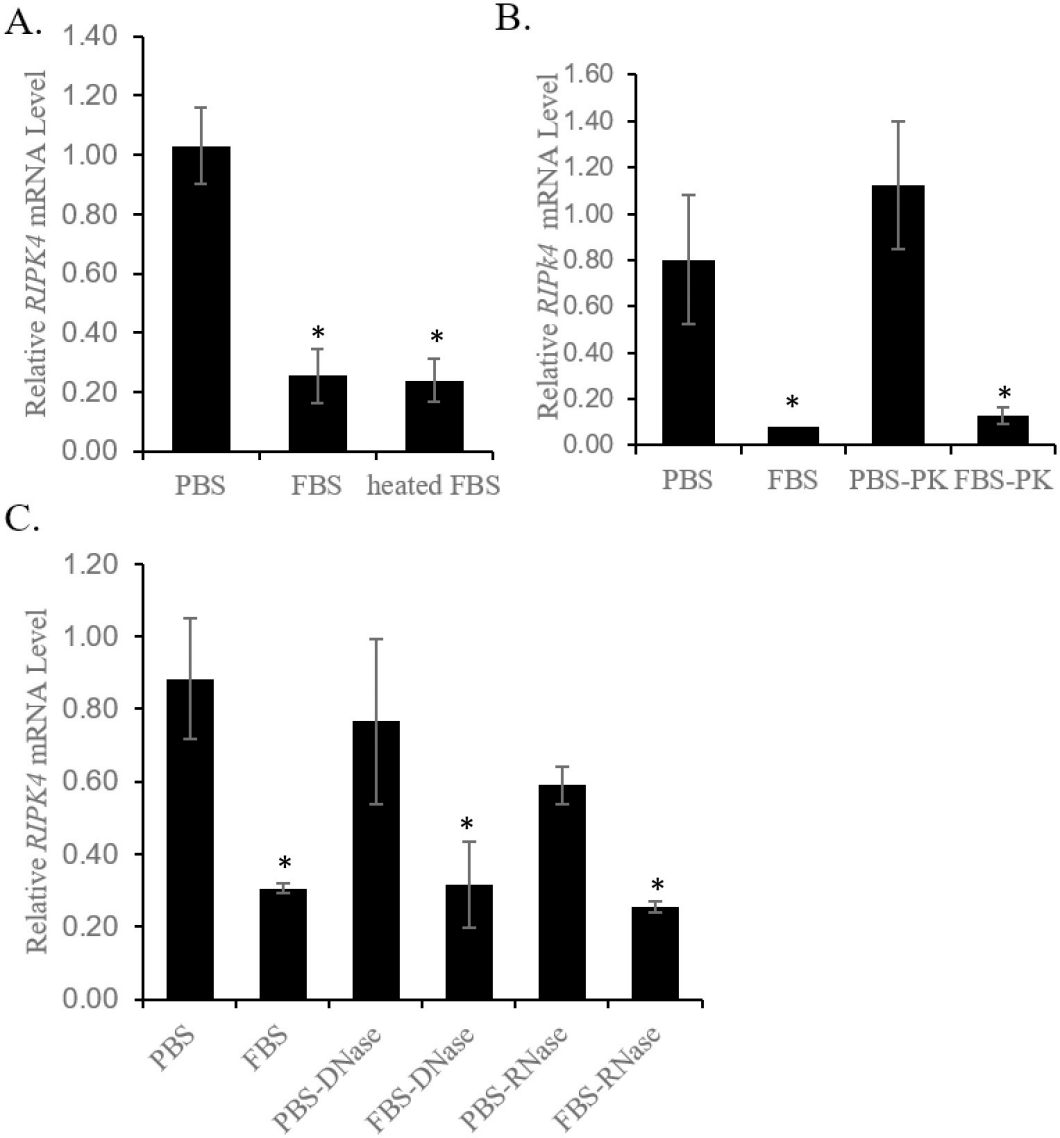
The serum factor that down-regulates *RIPK4* mRNA is unlikely a protein or DNA or RNA. A. Heated FBS retained its ability to down-regulate *RIPK4* mRNA in HaCaT cells. Data were pooled from two independent experiments. B. Proteinase K (PK) treatment did not abolish the ability of FBS to down-regulate *RIPK4* mRNA in HaCaT cells. C. DNase- or RNase-treatment did not affect the down-regulation of *RIPK4* mRNA in A431 cells by FBS. *: *p* < 0.05.

The major components in serum are proteins. We reasoned that if the serum factor is a protein, it would be likely sensitive to heating and protease digestion. Nevertheless, heating FBS at 95°C for 1hr did not abolish its ability to down-regulate *RIPK4* mRNA in HaCaT cells (Fig 3A); neither did overnight digestion by proteinase K (Fig 3B). Serum also contains extracellular DNA and RNA (for example microRNA), which may serve as the serum factor. Digestion of FBS by DNase I or RNase A did not abolish its effects on *RIPK4* mRNA in HaCaT cells (Fig 3C), suggesting that the serum factor is unlikely DNA or RNA.

**Fig 4:**
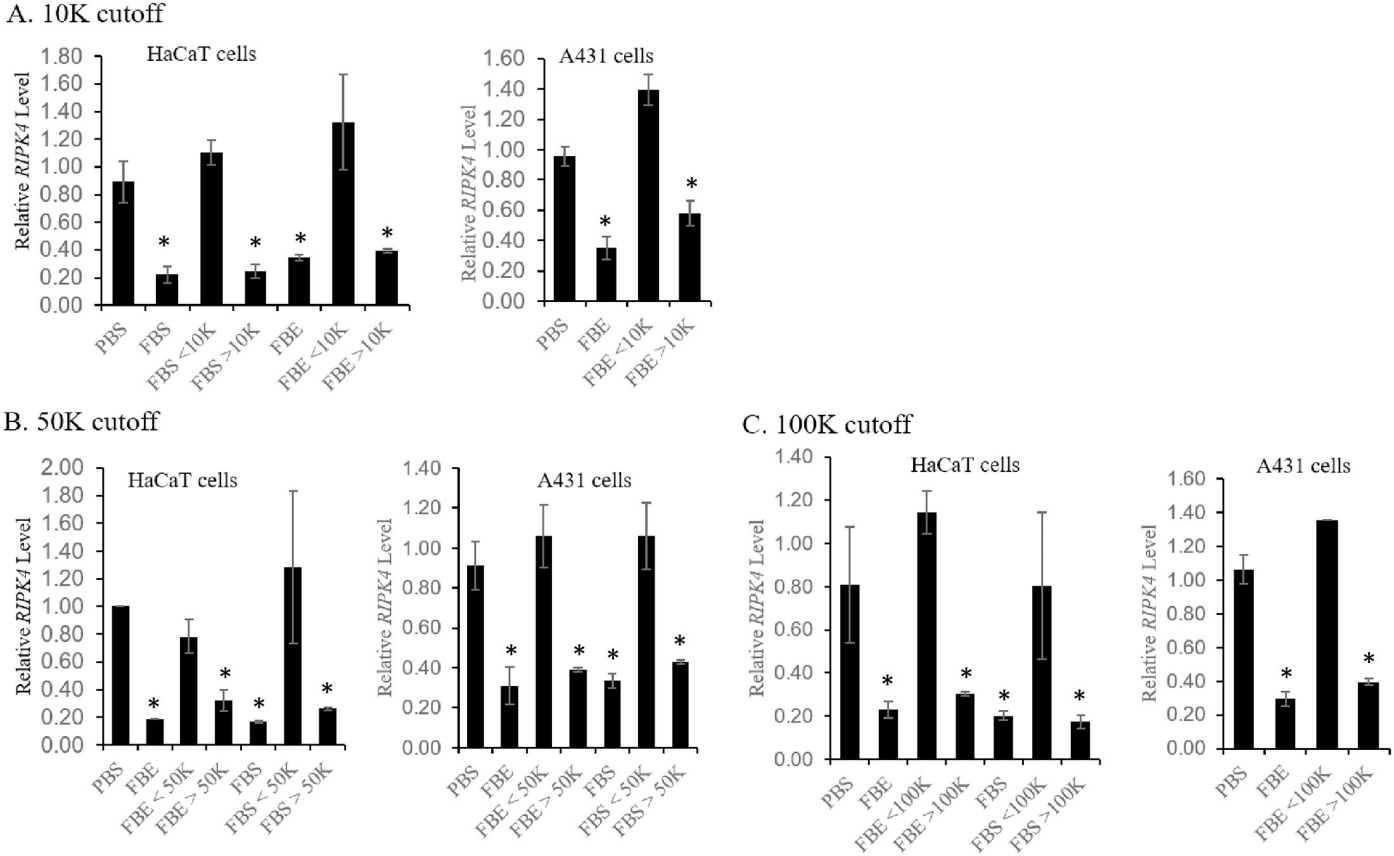
Size estimation of the serum factor. FBS or FBE was loaded onto centrifugal filtration columns with different cutoff of molecular weights: 10KDa (A), 50KDa (B), or 100KDa (C) (see Materials and Methods). Fractions retained by the columns should contain molecules larger than the cutoff and those flown through contain molecular smaller than the cutoff. Their activity to down-regulate *RIPK4* mRNA was tested on serum-starved monolayers of HaCaT or A431 cells and shown. See text for details.

We subsequently estimated the molecular weight (MW) of the serum factor. FBS or FBE was segregated by centrifugal filter columns into two fractions based on specific MW cutoff: one with smaller MW and the other with larger MW. The smaller molecules are supposedly in the flow-through and larger molecules are retained in the column. Filter columns with 10KD, 50KD, and 100KD MW cutoffs were sequentially analyzed. In each case, the retained fraction that includes molecules larger than the cutoff (i.e., >10K, >50K, or >100K) was found to down-regulate the level of *RIPK4* mRNA in both HaCaT and A431 cells, but not the flow-through that includes molecules smaller than the cutoff (Fig 4A–4C). These findings suggest that the serum factor may be a molecule larger than 100KD.

### BSA down-regulates *RIPK4* mRNA in keratinocyte-derived cell lines via its associated fatty acid LPA

**Fig 5:**
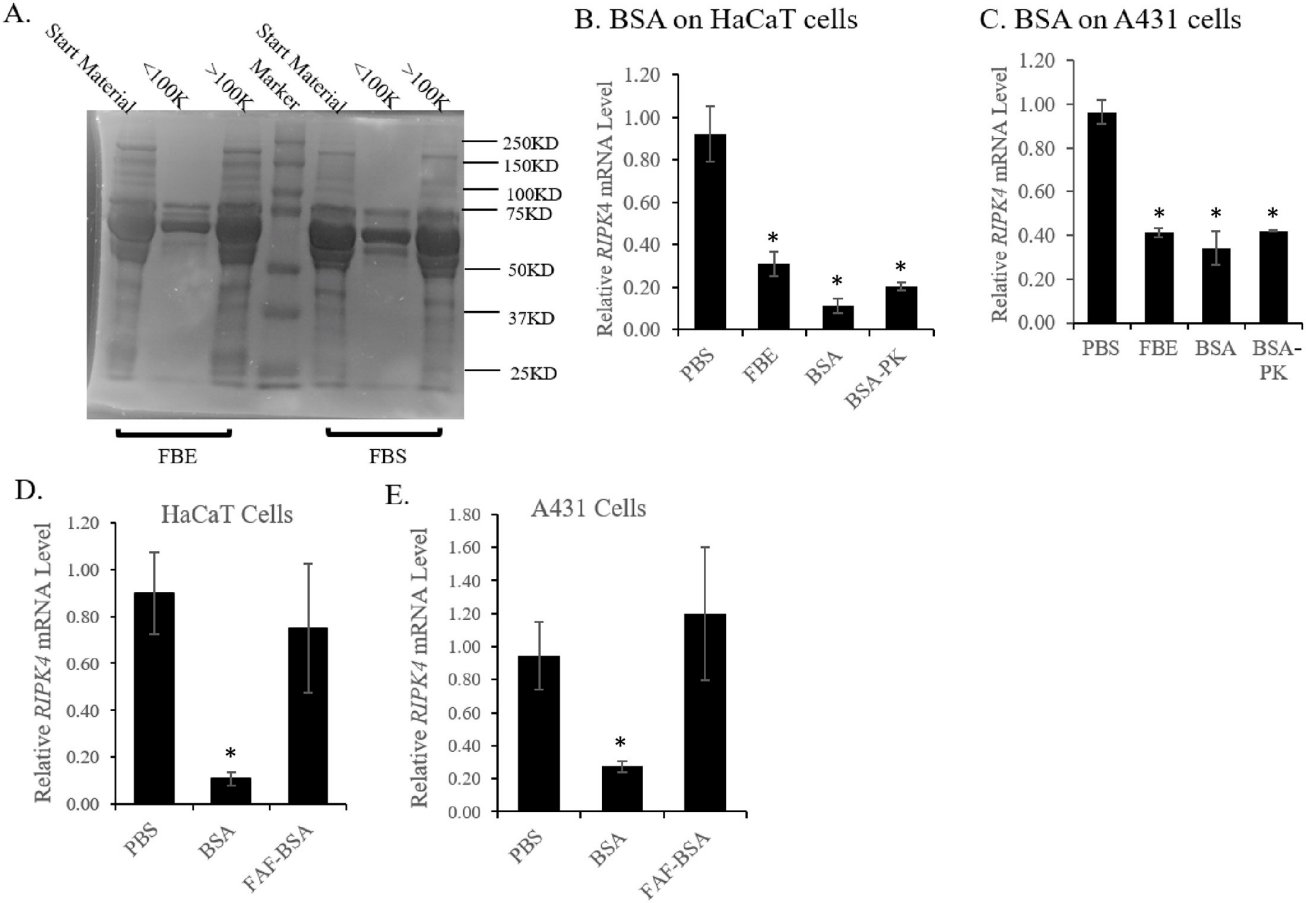
BSA but not fatty acid-free BSA (FAF-BSA) induced down-regulation of *RIPK4* mRNA in HaCaT and A431 cells. A. Fractions of FBS or FBE separated by columns with 100KD cutoff were analyzed on SDS-polyacrylamide gel. The majority of BSA was retained in the fractions larger than 100KD. B, C) 0.5% BSA induced down-regulation of PKK mRNA in HaCaT cells (B) or A431 cells (C), similar to FBE. This down-regulation was not abolished by proteinase K (PK) treatment. Data in B were pooled from two independent experiments. D, E) In contrast to BSA, 0.5% Fatty acid-free BSA (FAF-BSA) failed to suppress *RIPK4* mRNA expression in either HaCaT (D) or A431 (E) cells. Data in D were pooled from three independent experiments and data in E from two independent experiments. *: *p* < 0.05.

We examined the protein contents of fractions from the 100KD filters on SDS-PAGE and found that the predominant protein band in the >100K fraction corresponded to bovine serum albumin (BSA) (Fig 5A). BSA in its free form is about 66KD and so should be in the flow through from the 100KD filter unit but not in the >100K fraction. Perhaps BSA in serum binds other molecules in complexes, which are larger than 100KD, and/or its tertiary structure prevents its exclusion from the 100KD-cutoff column.

BSA is a major component in FBS [25] and, as shown above, stayed a dominant protein component in the fraction that down-regulated *RIPK4* mRNA. To isolate the serum factor, we decided to first exclude the possibility that BSA is the serum factor or carries the serum factor. 0.5% BSA was added to serum-starved HaCaT or A431 cells and, unexpectedly, it was found to down-regulate *RIPK4* mRNA at a level equivalent to FBS or FBE (Fig 5B and 5C). Furthermore, similar to FBS, proteinase K-treatment of BSA did not abolish its inhibitory effects (Fig 5B and 5C), suggesting that BSA may carry a serum factor and this factor is not its protein core. Since our previous studies indicated that this factor may not be DNA or RNA (Fig 3C), we predicted that it might be a lipid-related molecule. To test this, we obtained fatty acid-free BSA (FAF-BSA) and tested its effects on *RIPK4* mRNA expression. Consistent with our prediction, FAF-BSA failed to down-regulate *RIPK4* in either HaCaT or A431 cells (Fig 5D and 5E).

**Fig 6:**
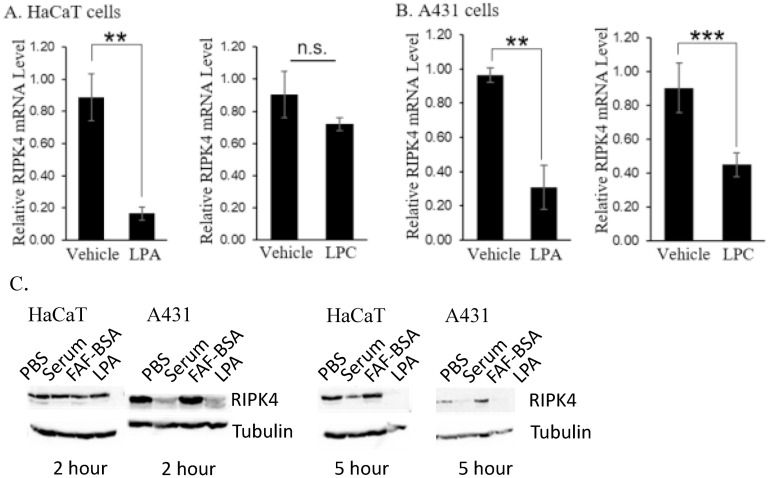
Lysophosphatidic acid (LPA) induced down-regulation of *RIPK4* in HaCaT and A431 cells. Serum-starved HaCaT (A) or A431 (B) cells were treated with 10 μM LPA or LPC for two hours. The level of PKK mRNA in these cells was measured and compared with vehicle control. The vehicle for LPA was FAF-BSA and for LPC ethanol. ***: *p* < 0.0005; ****: *p* < 0.00005, Student’s t test. n.s: not significant. Data for LPA treatment was combined from six independent experiments for HaCaT cells, and seven independent experiments for A431 cells. Data for LPC treatment were combined from four independent experiments for either cell line. HaCaT and A431 cells treated with serum or 10 μM LPA expressed decreased RIPK4 protein levels compared to control (C).

Albumin is a major carrier of lipids/fatty acids in serum [16, 26]. Among the fatty acids bound to albumin, a dominant species is lysophosphatidic acid (LPA) [16, 17, 26]. LPA signals through specific receptors on the cell surface and regulates a variety of important biological processes, including cell proliferation, migration, angiogenesis, and fibrosis [27]. We tested whether purified LPA induces the down-regulation of *RIPK4* in human keratinocyte cell lines. The concentration of LPA in serum is typically around 10 μM [27], so 10 μM LPA was added onto serum-starved HaCaT and A431 monolayers and found to down-regulate *RIPK4* mRNA (Fig 6A and 6B, left panels) to a similar extent as serum or BSA (Fig 5). Similar down-regulation was observed in human keratinocytes (S1B Fig, right). In contrast, its close structural analog, lysophosphatidylcholine (LPC), failed to induce *RIPK4* knockdown in HaCaT cells (Fig 6A, right panel), although it did induce some down-regulation of *RIPK4* in A431 cells (Fig 6B, right panel). Treatment with LPA led to decreased RIPK4 protein levels after 5 hours in both A431 and HaCaT cells (Fig 6C).

### *RIPK4* mediates the effects of LPA on cell cycle progression in HaCaT and A431 cells

**Fig 7:**
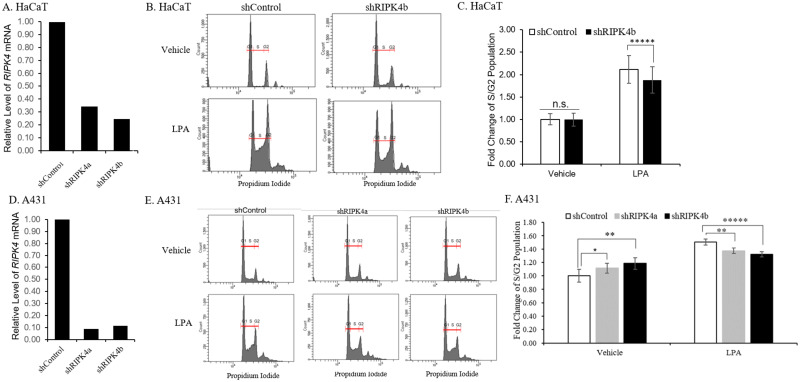
Depletion of *RIPK4* mediates the effects of LPA on cell cycle progression in HaCaT and A431 cells. A: *RIPK4* was knocked down by two shRNAs in HaCaT cells. *RIPK4* mRNA levels in the knockdown cells and control cells are shown; B: Cell cycle progression of control and *RIPK4*-knockdown HaCaT cells with or without LPA treatment was analyzed via propidium iodide staining and flow cytometry G1, S, and G2 populations were gated as indicated; C: The percentage of S/G2 population in control or *RIPK4* knockdown cells was compared after LPA or vehicle (FAF-BSA) treatment. *****: *p* < 0.00005, student’s t test; n.s: not significant. D: *RIPK4* was knocked down by two shRNAs in A431 cells. *RIPK4* mRNA levels in the knockdown cells and control cells are shown; E: Cell cycle progression of control and *RIPK4*-knockdown A431 cells with or without LPA treatment was analyzed via propidium iodide staining and flow cytometry; F: The percentage of S/G2 population in control or *RIPK4* knockdown cells was compared after LPA or vehicle (FAF-BSA) treatment. *: *p* < 0.05; **: *p* < 0.005; *****: *p* < 0.00005, student’s t test. Data were pooled from two independent experiments, with each condition tested in quadruplicate. Future studies will provide pathway analysis to define these pathways.

LPA is known to promote cell cycle progression in a variety of cell lines [28–31]. We have previously shown that down-regulation of *RIPK4* led to enhanced cell proliferation in keratinocyte- or SCC-derived cell lines [11]. Because LPA induced down-regulation of *RIPK4* in multiple keratinocyte-derived cell lines (Fig 6), we hypothesized that the down-regulation of *RIPK4* mediates the effects of LPA on cell proliferation. If this is the case, in the absence of *RIPK4*, the effects of LPA on cell cycle progression would be compromised. To test this, we knocked down *RIPK4* in HaCaT and A431 cells by shRNA (Fig 7A and 7D; S4 Fig) and analyzed the cell cycle progression of these knockdown cells upon treatment by LPA, in comparison with the control cells (Fig 7B and 7E). We found that LPA promoted cell cycle progression in both control and knockdown cells, compared with the vehicle control (FAF-BSA) (Fig 7C and 7F, left part of graph), but its effects on *RIPK4* knockdown cells were reduced compared with control (Fig 7C and 7F, right part of graph). Similar outcomes were observed when MTT analyses were performed, in which the differences between shControl and shRIPK4 cells were decreased upon LPA treatment, in comparison with vehicle control (S5 Fig). These findings suggest that down-regulation of *RIPK4* mediates at least some of the effects of LPA on cell proliferation.

## Discussion

We have previously shown that RIPK4 is down-regulated in SCCs and functions as a tumor suppressor in skin keratinocytes [10, 11]. In the current study, we identified a factor that mediates this down-regulation and it is a phospholipid, lysophosphatidic acid (LPA). We consider our finding a breakthrough in understanding RIPK4 biology. LPA is an abundant phospholipid that plays pleotropic roles in a variety of important biological processes, including cancer, neurological disorder, angiogenesis, and wound healing [27]. It is most abundant in serum, where it binds to serum albumin and can be delivered to skin keratinocytes during differentiation, carcinogenesis, and wound healing [31]. It is also produced in resident tissues and its level is often found elevated under pathological conditions [18–20]. It is thus possible that RIPK4 in keratinocytes is down-regulated by LPA in serum, which is released during wounding or carcinogenesis, or by LPA from resident tissues during pathophysiological processes; and this down-regulation leads to elevated proliferation of keratinocytes. Indeed, our current study and other reports [29, 30] showed that LPA can drive the proliferation of keratinocyte-derived cell lines and LPA has been suggested to be a tumor promoter in esophageal squamous cell carcinomas, colorectal cancers, glioblastoma, ovarian cancers, and other cancers [28, 32–35]

Our earlier study showed that the loss of RIPK4 in skin SCCs led to enhanced cell proliferation, probably due to upregulation of nuclear p63 and decreased NF-kB pathway activation [10, 11]. These outcomes of RIPK4 loss may explain, at least partly, the proliferative effects of LPA on keratinocyte-derived cell lines, since those effects were compromised in *RIPK4*-knocked down cell lines (Fig 7). As mentioned above, RIPK4 knockdown in keratinocyte-derived cells had been shown to promote proliferation [10, 11]. In those studies, cells were growing continuously in serum-containing medium without going through the starvation-to-serum stimulation process, so the control cells maintain their expression of RIPK4 and exhibit reduced proliferation than the RIPK4-knockdown cells. In our study, LPA treatment after a starvation period induced the down-regulation of RIPK4 in control cells, which would be equivalent to the RIPK4-knockdown cells in proliferation. Nevertheless, our results suggest that the RIPK4-knockdown cells may be less proliferative than control cells in the presence of LPA (Fig 7C and 7F). One possibility is that LPA induces additional changes in control cells to promote their proliferation and these changes do not occur as effectively in RIPK4-knockdown cells. Alternatively, transient knockdown of RIPK4 induced by LPA is more potent than the stable knockdown in stimulating proliferation. Which or both of these mechanisms occur in LPA/RIPK4-regulated cell proliferation in keratinocytes await further investigations. It is worth noting that the impact of *RIPK4* knockdown on LPA-induced proliferation was not drastic, although statistically significant. This could be because LPA signaled through parallel pathways to promote cell proliferation [27, 29], and/or LPA induced down-regulation of *RIPK4* in at least part of the 24-hour period we analyzed so the treated cells would be equivalent to *RIPK4*-knockdown cells during that period.

A variety of fatty acids are carried by albumin in serum [16, 26]. We tested only LPA and LPC in our study, partly because they are among the most abundant fatty acids on albumin [16, 17]. LPA also exists in many isoforms [18]. The 18:1 oleoyl isoform used in our study is the most commonly studied and readily commercially available. It had also been shown to exhibit the same level of activity as all phospholipids extracted from albumin [36]. LPC serves as a precursor of LPA but could also signal on its own *in vivo* [17]. Because of its structural similarity to LPA but distinct signaling characteristics, it has frequently been used as a control phospholipid in biological assays [37]. In our study, LPC did not impose a similar effect on down-regulating *RIPK4* mRNA in HaCaT cells as LPA (Fig 6A), but it did in A431 cells (Fig 6B). It is possible that in A431 cells, LPC could be efficiently converted to LPA and so as to exert down-regulating effects on *RIPK4*. The conversion of LPC to LPA is catalyzed by an enzyme called Autotaxin (ATX) [18, 38]. ATX is upregulated in many cancer types compared with normal tissues [38]. A431 is a tumorigenic SCC cell lines whereas HaCaT is non-tumorigenic. It is thus conceivable that A431 cells express higher levels of ATX and as such converts LPC to LPA more efficiently than HaCaT cells. Alternatively, A431 cells may express the receptor for LPC to enable its signaling but HaCaT cells do not.

The connection we revealed between LPA and RIPK4 inhibition would provide insight not only on RIPK4-regulated cancer progression but also facilitate understanding other aspects of RIPK4-related biology. As mentioned earlier, *ripk4* mRNA was found down-regulated in mouse skin during wounding [15]. Our finding suggests that this down-regulation may be due to signaling from LPA, which is abundant in wounds and whose role in inflammation and wounding had been well documented [27]. LPA has been shown to promote re-epithelialization in excisional skin wound healing models [39, 40]. This supportive role may be mediated by loss of RIPK4. Further defining the pathways important for LPA’s effect on RIPK4 and ultimately whether LPA’s control over RIPK4 can promote wound healing will be an area of future work that could lead to new treatments for acute and chronic wounds.

## Supporting information

S1 Fig Scratching did not affect the level of *RIPK4* mRNA in HaCaT cells in the presence of FBS and FBS or LPA treatment down-regulated *RIPK4* mRNA in human adult keratinocytes (HEKa). A. A scratch was introduced on serum-starved HaCaT cells by a pipet tip and cells were then treated with FBS. RNA was harvested 0, 2, or 4 hours later and the level of RIPK4 mRNA was measured and compared with samples without scratching. B. HEKa cells were treated with 10% FBS or 10 μM LPA for 2 hours. Each treatment was performed in duplicate and quadruplicate in two independent experiments. The level of RIPK4 mRNA was measured and found reduced in the FBS- or LPA-treated samples, compared with controls.
(TIF)

S2 Fig Similar down-regulation of *RIPK4* by FBS was observed when an alternative pair of primers was applied in the quantitative RT-PCR reaction. Serum-starved A431 monolayer was treated with FBS or PBS for two hours and RNA was harvested. *RIPK4* mRNA level was measured using an alternative pair of primers (see Materials and Methods). *: *p* < 0.05.
(TIF)

S3 Fig Actinomycin D treatment depleted *RIPK4* mRNA in HaCaT cells. HaCaT monolayer was treated with 10μg/ml Actinomycin D (ActD) or PBS for three hours. RNA was harvested and the level of *RIPK4* measured. *: *p* < 0.05.
(TIF)

S4 Fig shRNA knockdown led to reduced levels of RIPK4 protein in HaCaT and A431 cells. HaCaT (A) or A431 cells (B) expressing control shRNA or shRNAs against RIPK4 were lysed in homogenization buffer and probed by rabbit anti-RIPK4 antibody (Cell signaling #12636) and mouse anti-tubulin antibody (Sigma #T8328) on western blots.
(TIF)

S5 Fig MTT analysis on HaCaT cells expressing shControl and shRIPK4. HaCaT cells expressing control shRNA or shRNA against RIPK4 were analyzed for cell proliferation after LPA or vehicle treatment by MTT assay. Whereas enhanced cell proliferation was observed in RIPK4-knockdown cells after vehicle treatment, this difference was minimized when LPA was administered. *: *p* < 0.05; ****: *p* < 0.00005, Student’s t test.
(TIF)

S1 Raw image (TIF)

S2 Raw image (TIF)

S3 Raw image (TIF)

S4 Raw image (TIF)
