# Supplementary figures and images for: Lysophosphatidic acid down-regulates human RIPK4 mRNA in keratinocyte- derived cell lines
Source: PLoS One. 2024 Apr 17;19(4):e0287444. doi: 10.1371/journal.pone.0287444 (PMC11023271; doi:10.1371/journal.pone.0287444)

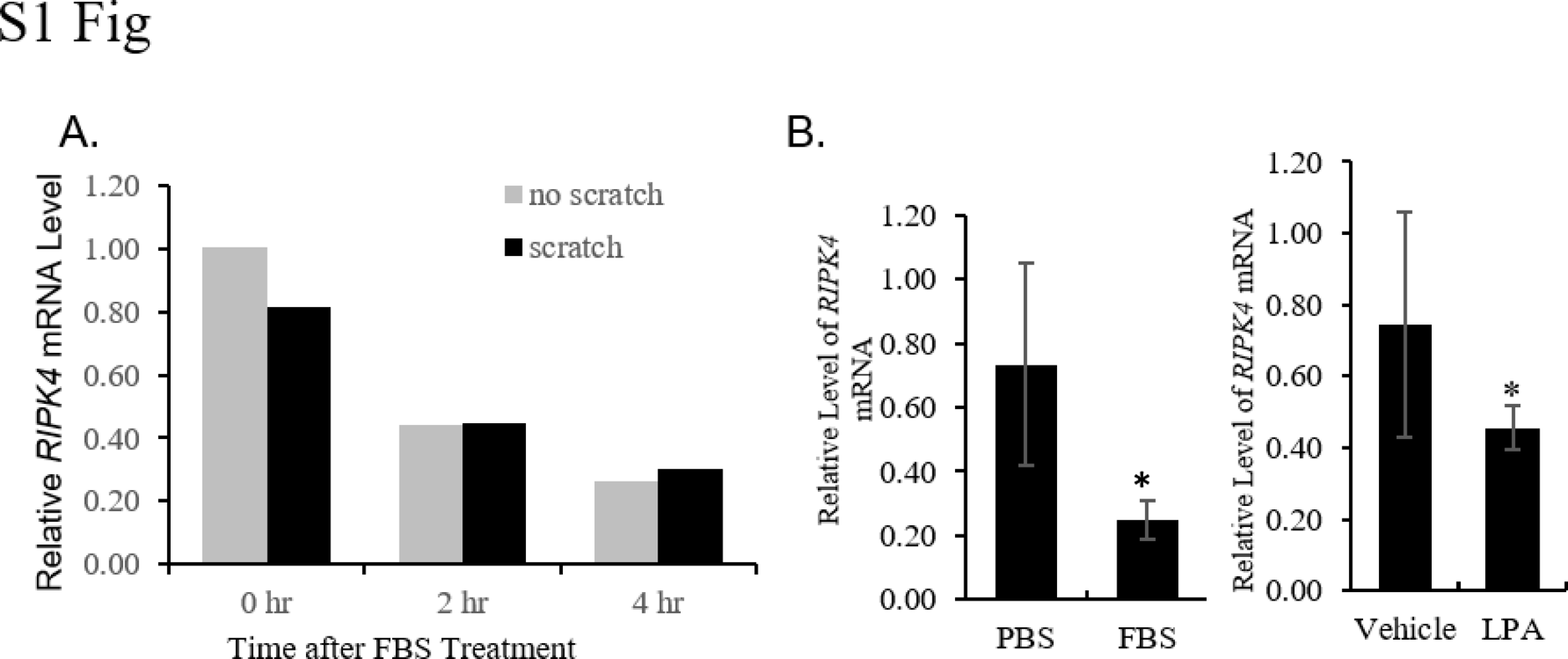

Supplement: S1 Fig — A. A scratch was introduced on serum-starved HaCaT cells by a pipet tip and cells were then treated with FBS. RNA was harvested 0, 2, or 4 hours later and the level of RIPK4 mRNA was measured and compared with samples without scratching. B. HEKa cells were treated with 10% FBS or 10 μM LPA for 2 hours. Each treatment was performed in duplicate and quadruplicate in two independent experiments. The level of RIPK4 mRNA was measured and found reduced in the FBS- or LPA-treated samples, compared with controls. (TIF) [file pone.0287444.s001.tif]

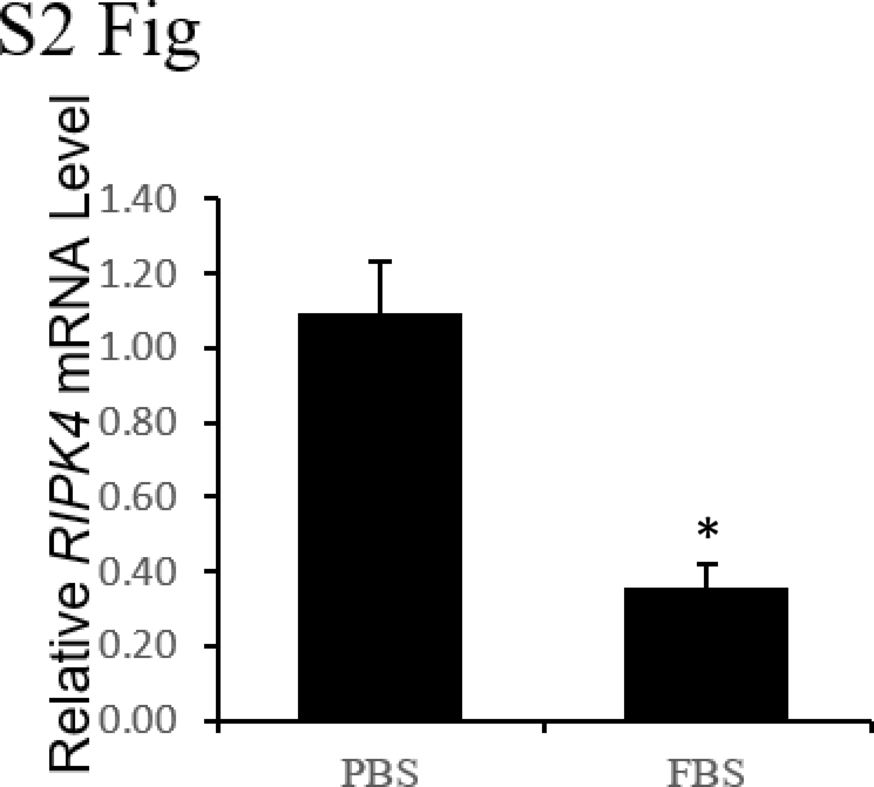

Supplement: S2 Fig — Serum-starved A431 monolayer was treated with FBS or PBS for two hours and RNA was harvested. RIPK4 mRNA level was measured using an alternative pair of primers (see Materials and Methods). *: p < 0.05. (TIF) [file pone.0287444.s002.tif]

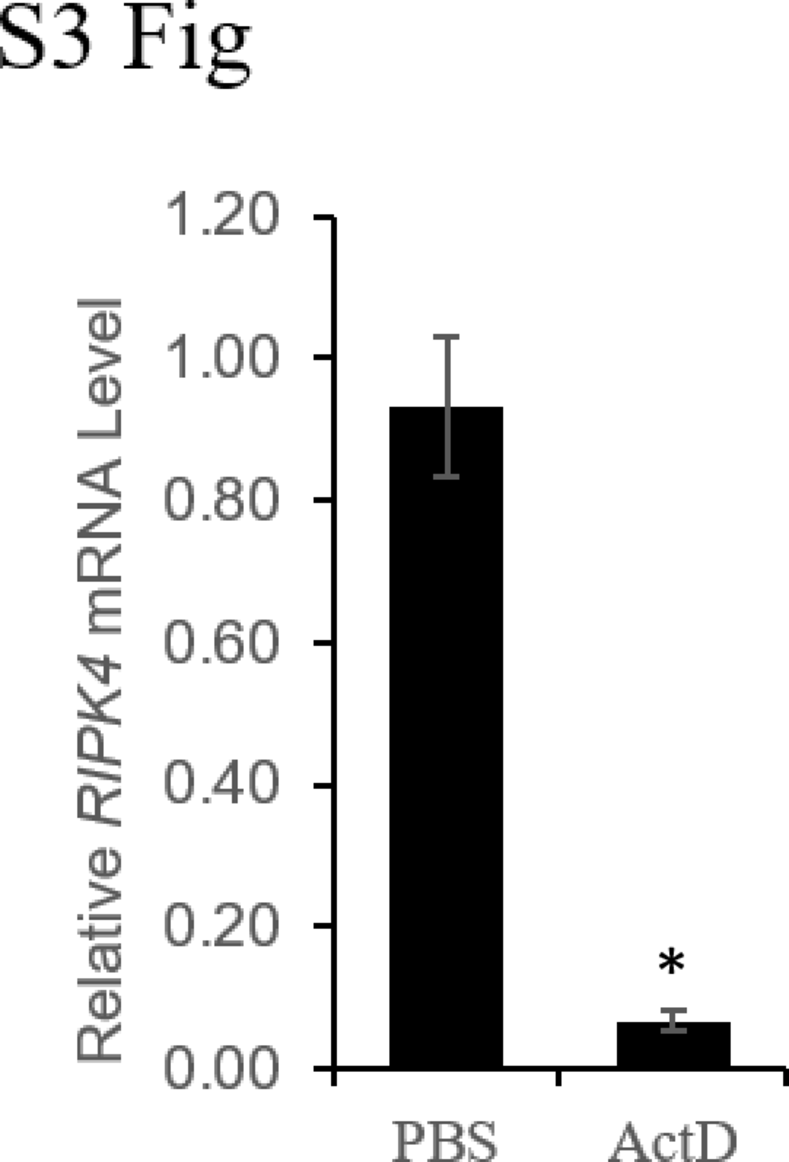

Supplement: S3 Fig — HaCaT monolayer was treated with 10μg/ml Actinomycin D (ActD) or PBS for three hours. RNA was harvested and the level of RIPK4 measured. *: p < 0.05. (TIF) [file pone.0287444.s003.tif]

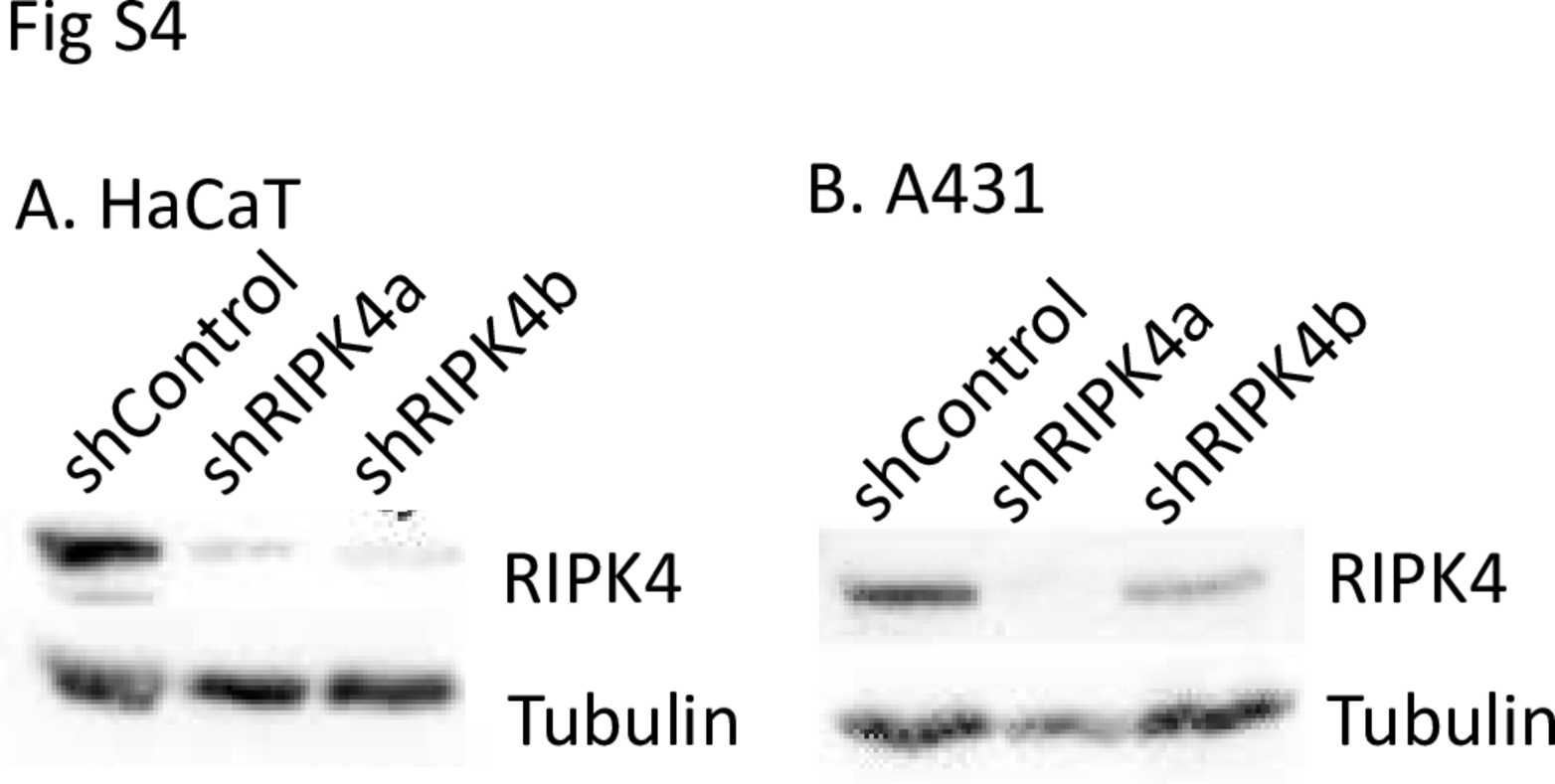

Supplement: S4 Fig — HaCaT (A) or A431 cells (B) expressing control shRNA or shRNAs against RIPK4 were lysed in homogenization buffer and probed by rabbit anti-RIPK4 antibody (Cell signaling #12636) and mouse anti-tubulin antibody (Sigma #T8328) on western blots. (TIF) [file pone.0287444.s004.tif]

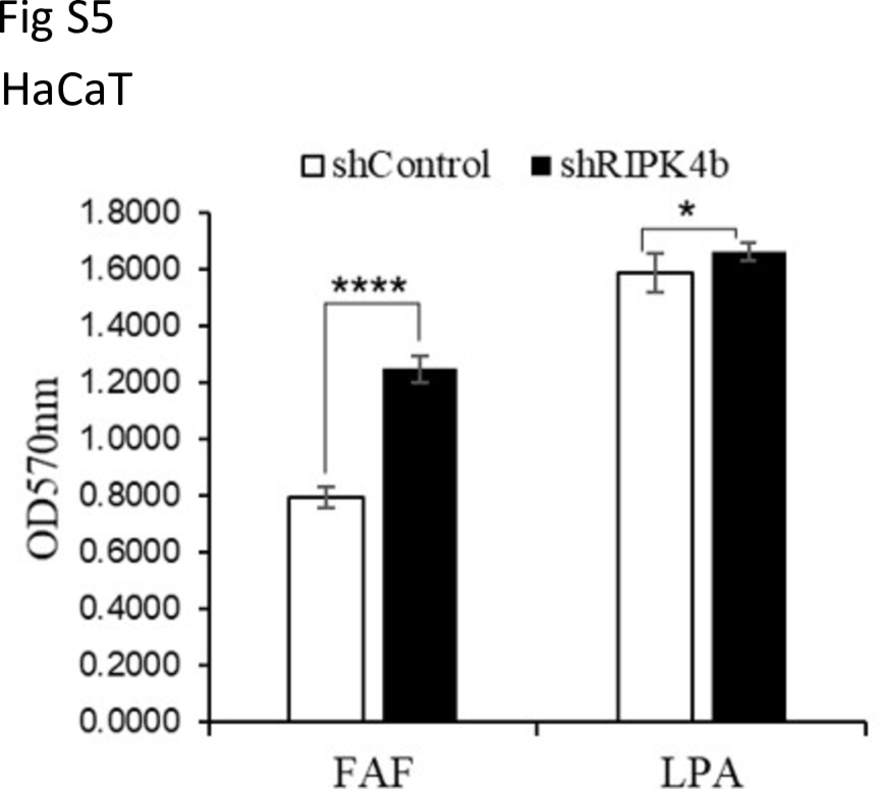

Supplement: S5 Fig — HaCaT cells expressing control shRNA or shRNA against RIPK4 were analyzed for cell proliferation after LPA or vehicle treatment by MTT assay. Whereas enhanced cell proliferation was observed in RIPK4-knockdown cells after vehicle treatment, this difference was minimized when LPA was administered. *: p < 0.05; ****: p < 0.00005, Student’s t test. (TIF) [file pone.0287444.s005.tif]

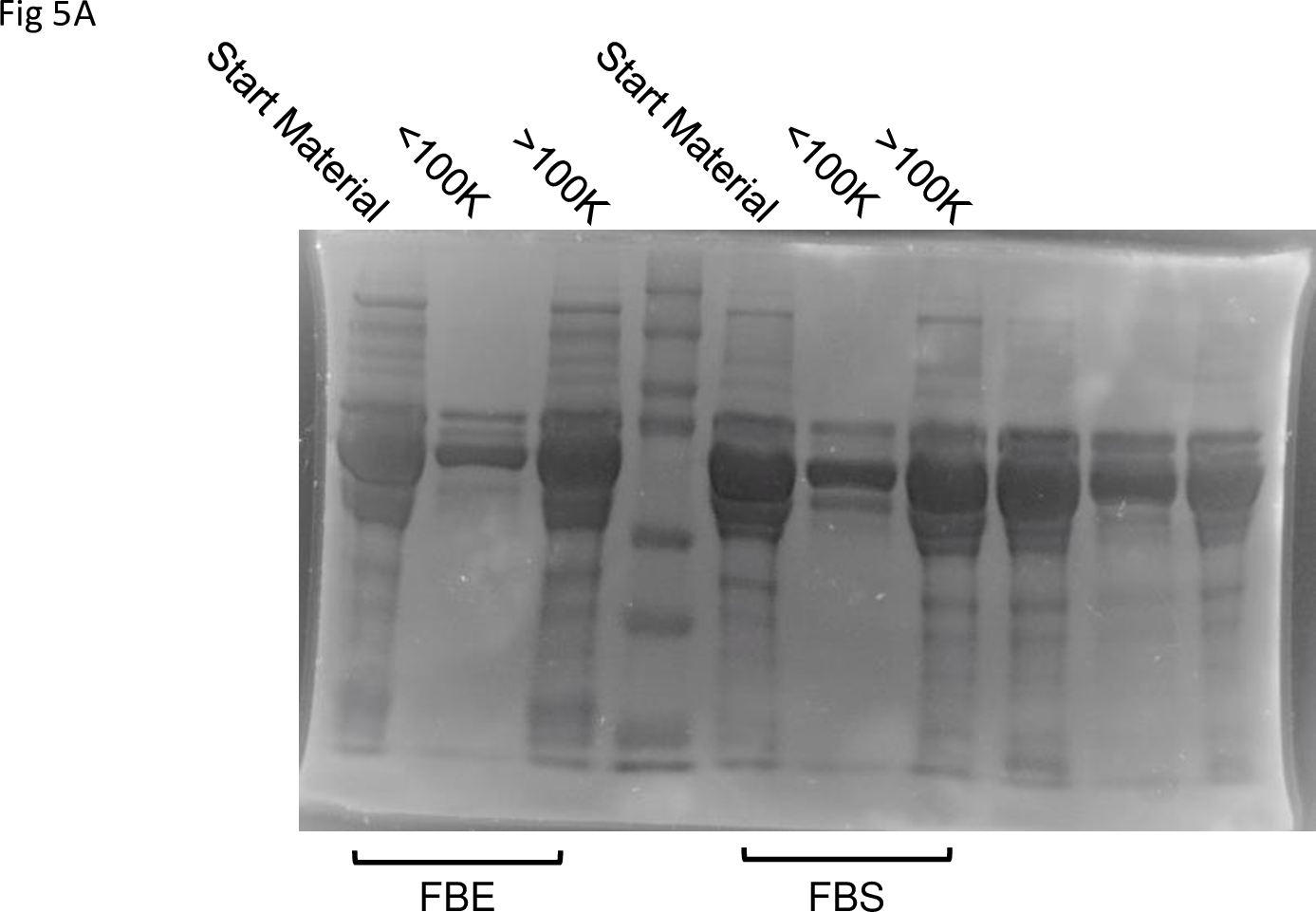

Supplement: S1 Raw image — (TIF) [file pone.0287444.s006.tif]

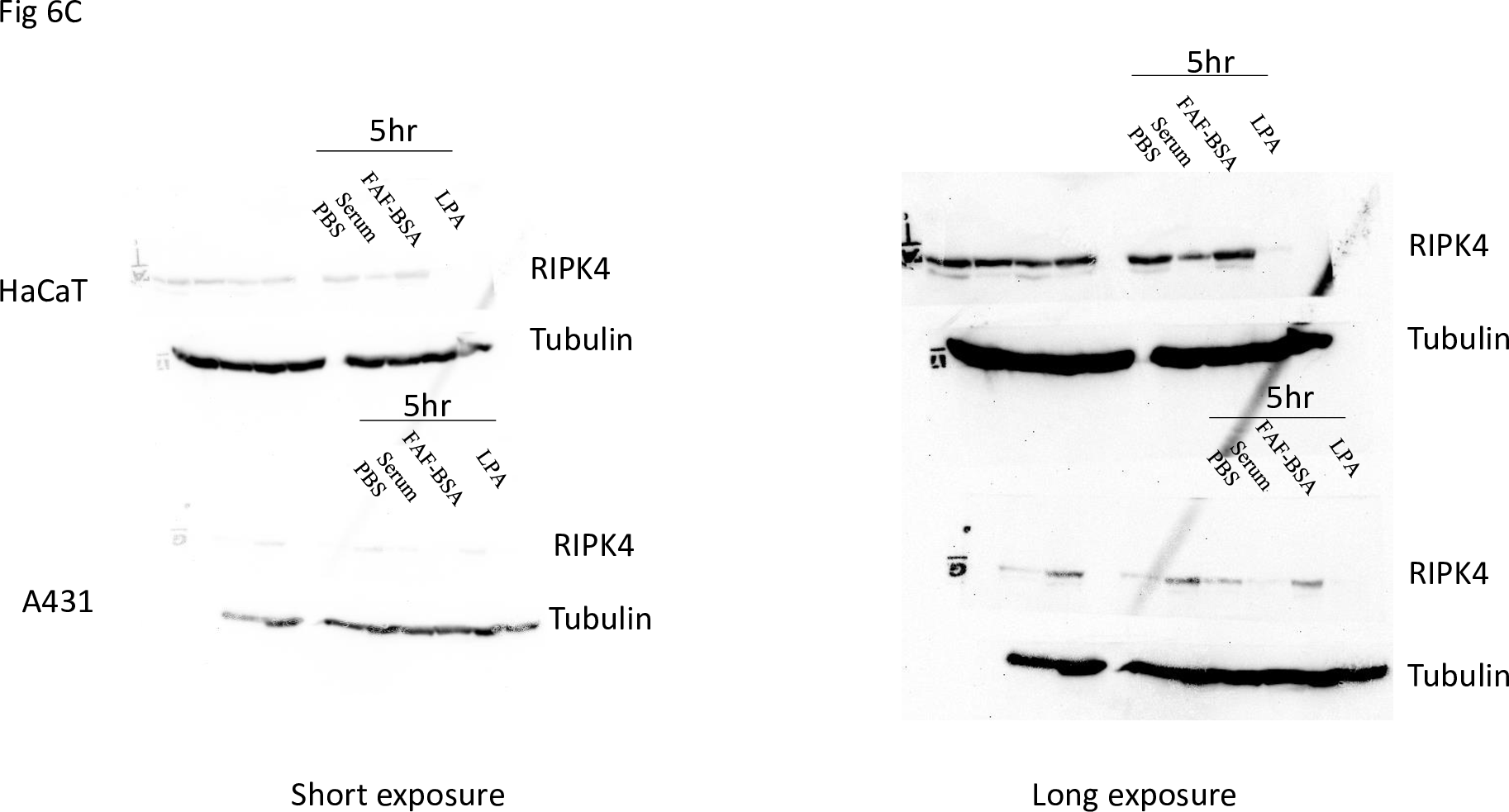

Supplement: S2 Raw image — (TIF) [file pone.0287444.s007.tif]

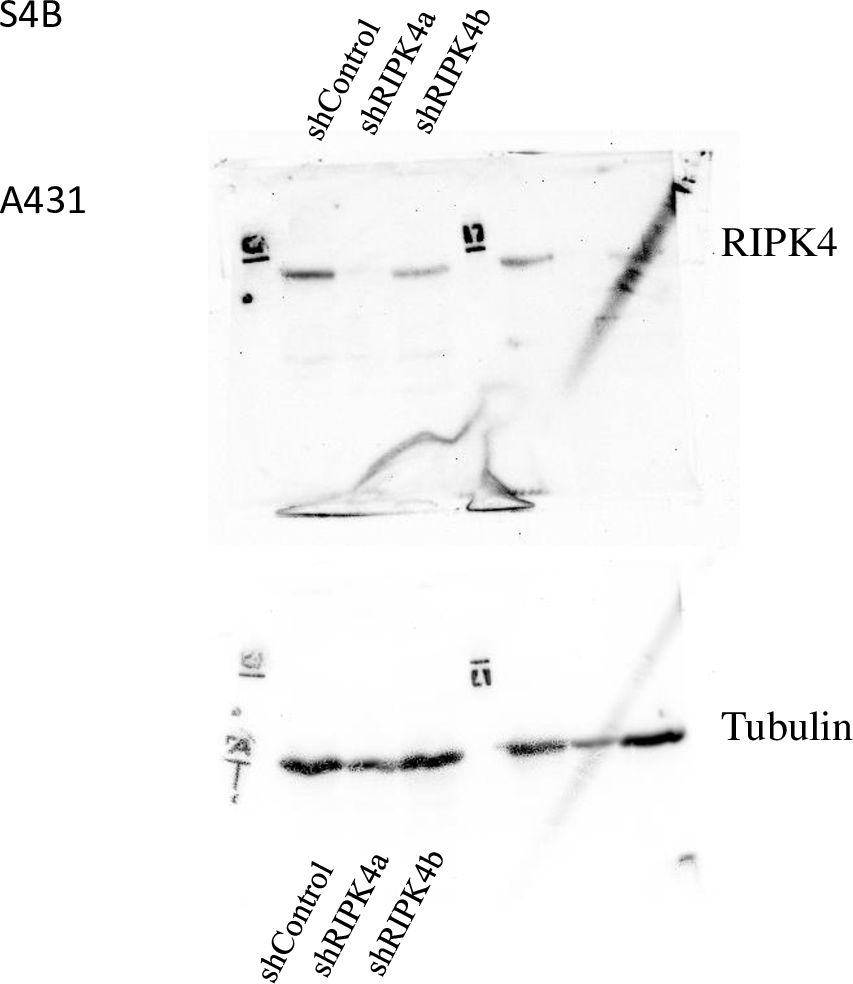

Supplement: S3 Raw image — (TIF) [file pone.0287444.s008.tif]

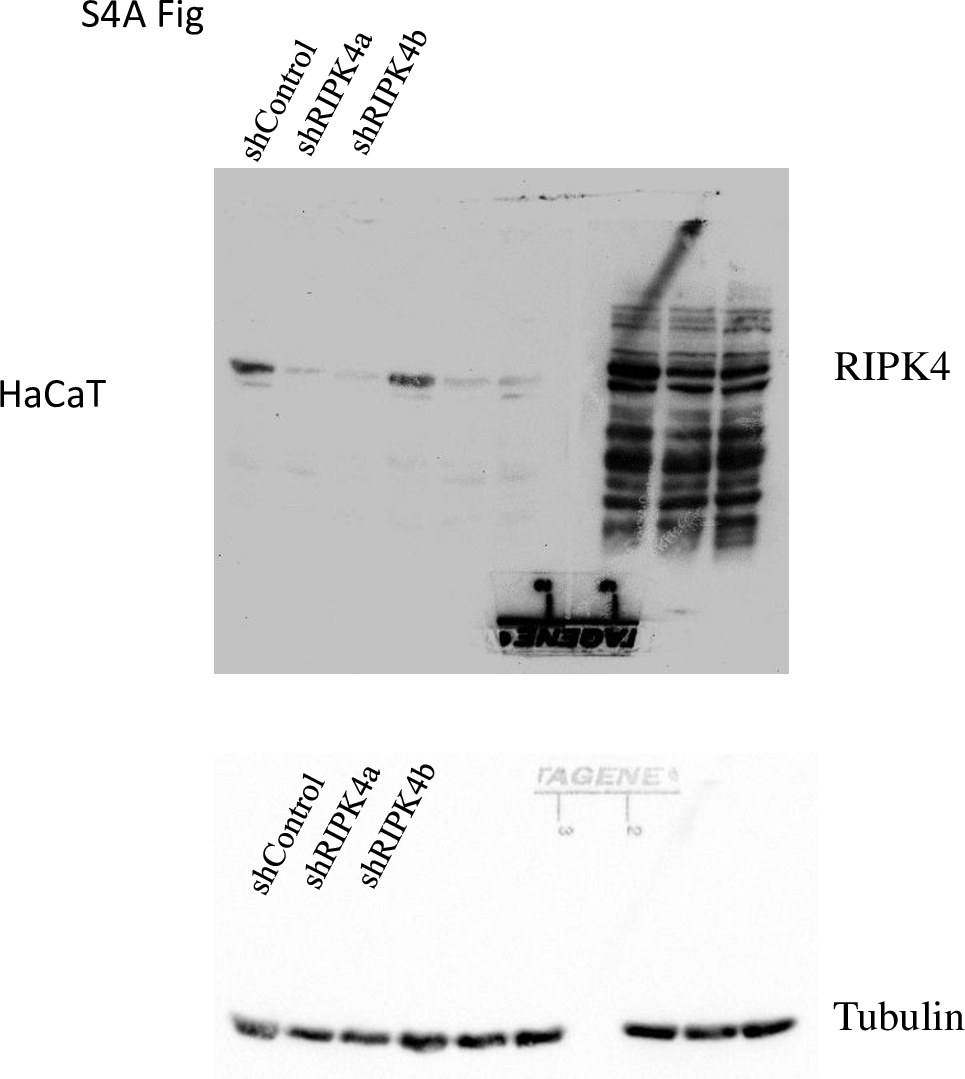

Supplement: S4 Raw image — (TIF) [file pone.0287444.s009.tif]
